# Analysis of drilling vibration characteristics of anchoring system in coal mine

**DOI:** 10.1038/s41598-023-46451-y

**Published:** 2023-11-07

**Authors:** Miao Xie, Yuqi Li, Hongyu Zhang, Zhiyong Yang, Ze Ren, Rendong Nie

**Affiliations:** 1 College of Mechanical Engineering, Liaoning Technical University, Fuxin, 123000 China; 2 National Energy Group International Engineering Consulting Co., Ltd, Beijing, 100010 China; 3 Xinjiang Key Laboratory of Intelligent Exploit and Control of Open-Pit Mine, Changji, 831100 China

**Keywords:** Mechanical engineering, Applied mathematics

## Abstract

A new type of parallel operation unit for excavating and supporting anchors is proposed to address the issue of imbalanced excavation anchor ratio in coal mines. By equipping a straddle type anchoring drilling rig group, the synchronous parallel and fast operation mode for excavating and supporting anchors is achieved; Consider the problem of poor drilling stability of drill pipes in coal mines due to the coupling vibration between surrounding rock and anchoring equipment. Firstly, taking the multi drilling rig anchoring system as the research object, considering the influence of the equipment itself as an influencing factor on the vibration of the drill pipe, a dynamic model of the system is constructed using Lagrangian equations, and analytical solutions for the vibration displacement of each mass block are obtained; In order to more intuitively represent the vibration process of the drill pipe, Ansys is used to conduct modal analysis on the key components of the anchoring drilling rig system, and obtain the natural frequencies and vibration modes of each order of the key components; Using Adams to solve the rigid flexible coupling dynamic model of the anchoring drilling rig system, the vibration response laws of the drill pipe under different operating states were obtained. Secondly, Abaqus was used to simulate the drilling process of the drill pipe and obtain the vibration response law generated by the interaction between the drill pipe and the surrounding rock; The results indicate that the anchoring equipment has a greater impact on the vibration of the drill pipe, and the surrounding rock has a more stable impact on the vibration of the drill pipe. Due to the short body and large span structure of the anchoring system crossbeam expansion frame, the vibration response of the drill pipe is significantly greater than that of the retracted state of the drilling rig due to being in an unstable cantilever state when the drilling rig is extended. The theoretical reliability of the vibration response law of the drill pipe under different states has been further verified through drilling experiments of the anchoring system prototype. The relevant theories can provide a theoretical basis for the implementation of automatic anchoring technology in the anchoring system.

## Introduction

The uneven development of coal seam joints leads to instantaneous loads during the cutting process, which is characterized by strong coupling, nonlinearity, and complexity. Analyzing the vibration characteristics of anchor drilling rigs and drill pipes plays an important role in studying the stability of anchor drilling rigs, achieving precise control of anchor drilling robots, predicting the physical properties of coal and rock masses, and improving the quality of anchor drilling^1–6^. Schumski et al. found that changing the vibration mode can significantly reduce the maximum thrust and torque^7^. Yang et al. established a time-domain vibration model of the center of gravity of the anchor rod drilling rig, obtained the vibration response signal of the drilling rig through Simulink simulation, and analyzed the stability of the working process of the anchor rod drilling rig^8^. Chen et al. used the Bekker model to describe the nonlinear behavior between the track of the excavation machine and the tunnel floor, and then established a multi degree of freedom nonlinear vibration equation for the excavation anchor combined machine based on Newton's kinematic law, analyzing the vibration characteristics of the entire machine^9^. Gopalkrishna et al. studied the optimal stable rest number of gun drilling rigs by establishing an empirical relationship between the maximum amplitude of resonance frequency and the length and diameter of the borehole^10^. Kondratenko et al. established a differential equation for drill pipe vibration based on the Lagrange equation and studied the variation of drill pipe drilling speed and stress at different lengths^11^. Wang et al. established a rigid flexible coupling dynamic model of the horizontal drilling rig drill pipe hole wall system based on the second type of Lagrange equation and the Newton Euler method, and found that the drilling depth has a greater impact on the system^12^. Nogueira and Ritto considered the effects of torsional excitation and mud on the system, modeled using the classical torsional theory of the finite element method, and obtained a random stability map using the Monte Carlo method^13^. Jun et al. solved the problem of dynamic model uncertainty caused by time-varying internal parameters and external loads in hydraulic servo systems, and optimized and reconstructed the structure and motion coefficient parameters of the robotic arm^14^. Liu et al. established a vibration transfer model for the joint subsystem of a six degree of freedom robotic arm based on multiple transfer path analysis (MTPA) and modal superposition method (MSM), and obtained the amplitude of each degree of freedom of the support^15^. Liu et al. can predict the position of rock interface by collecting displacement data during rock drilling and establishing drilling speed models and longitudinal vibration models^16^. Wang et al. established a theoretical model for the longitudinal vibration of end anchor bolts, solved the dynamic acceleration response of end anchor bolts under transient impact under different boundary conditions, and analyzed the parameters that affect the anchoring quality of anchor bolts based on this theoretical model^17^.

Some studies aim to better understand the overall dynamics of rotary drilling systems^18–20^. Stevenyi et al. proposed a two degree of freedom lumped parameter model that considers the coupling of axial and torsional vibrations and smoothes the control equation^21^. Melakhesou et al. established a nonlinear dynamic model for the longitudinal, transverse, and torsional coupling of the drill string system^22^. Li et al. established a classification model for vibration patterns using supervised machine learning methods. By applying the classification model to actual drilling data, the specific vibration types of each vibration mode were identified^23^. Khulef et al. established a finite element dynamic system considering gyroscope torque, solved the modal characteristics of the drill string, and solved the time response of the drill string system using Lagrangian and finite element methods^24^. Kovalyshen et al. established a simplified drill bit vortex analysis model that considers the effects of drill bit shape and drill bit rock contact^25^. Shan et al. established a finite element flexible simulation model for drill string dynamics and used a stored vibration measurement device to collect actual drilling vibration data, verifying the correctness of the simulation model^26^. Aadnoy et al. studied the friction and friction reduction between the drill string and wellbore during the tripping process and proposed a method of utilizing drill string rotation^27^. Xie et al. considered the uneven factors of the roof and floor of coal mine tunnels and studied the influence of different drilling angles and leg angles on leg force when multiple drilling rigs were drilling simultaneously^28^.

At present, few people have studied the vibration characteristics of parallel drilling systems with multiple anchor rods. For underground coal mines with complex geological conditions, using multiple anchor rod drilling machines in parallel can improve excavation efficiency^29–32^. Therefore, studying the vibration characteristics of multiple anchor rod drilling machines in parallel drilling systems is of great significance for achieving unmanned coal mine excavation. This article takes multi drill drilling as the research object, studies the vibration response law of drill pipes under the coupling effect of surrounding rock and anchoring system, analyzes the impact of anchoring system equipment vibration on drill pipe vibration, and analyzes the drill pipe vibration under the interaction between the drilling process and surrounding rock. Based on the Lagrange equation, a mathematical model of the anchoring system was established, and Adams and Abaqus solved the above model. Finally, the theoretical reliability of the drilling vibration law of the key components of the drilling rig was verified through the drilling test of the anchoring system prototype.

## Construction and solution of dynamic model of anchorage group

### Construction of dynamic model

**Figure 1:**
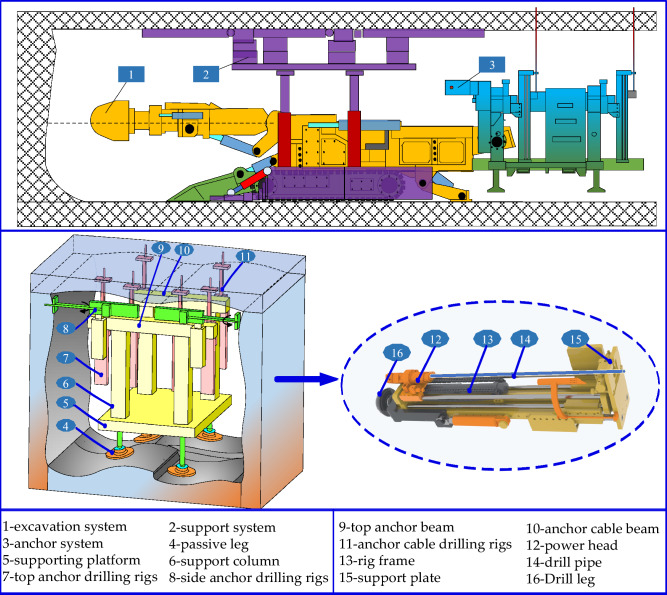
Overall diagram of anchorage system.

The anchoring system for coal mines can realize the function of following the machine. The roadheader is separated from the anchoring system during the tunneling operation, which can realize the simultaneous anchoring operation of multiple drilling rigs and improve the anchoring efficiency. The existing rapid excavation system has poor practical application effect in coal mines, mainly due to issues such as poor stability, low efficiency, and difficulty in following the machine during anchoring operations. In response to the existing problem of parallel excavation and anchor operation in the above comprehensive excavation face, a new type of fast excavation unit for excavation, support, and anchor transportation is proposed, which mainly consists of three parts: excavation machine, advanced support equipment, and anchoring group system, as shown in Fig. 1. The advanced support equipment adopts a step-by-step non repetitive rolling method, with multiple sets of flexible support units arranged above the support, which can adapt to temporary support operations under different geological conditions; the excavation system and anchoring group system can achieve a separation of straddles and achieve synchronous walking of excavation and anchoring. During the excavation operation, the excavation anchors are separated and do not interfere with each other, resulting in a compact system and high space utilization. As an important component of the new intelligent excavation system, the anchoring system is of great significance in studying its mechanical characteristics and reliability. The focus is on studying the vibration response law of the drill pipe under the dual influence of the machine body vibration and surrounding rock action during the drilling process, ensuring the safety and efficiency of the drilling process and improving the drilling efficiency.

**Figure 2:**
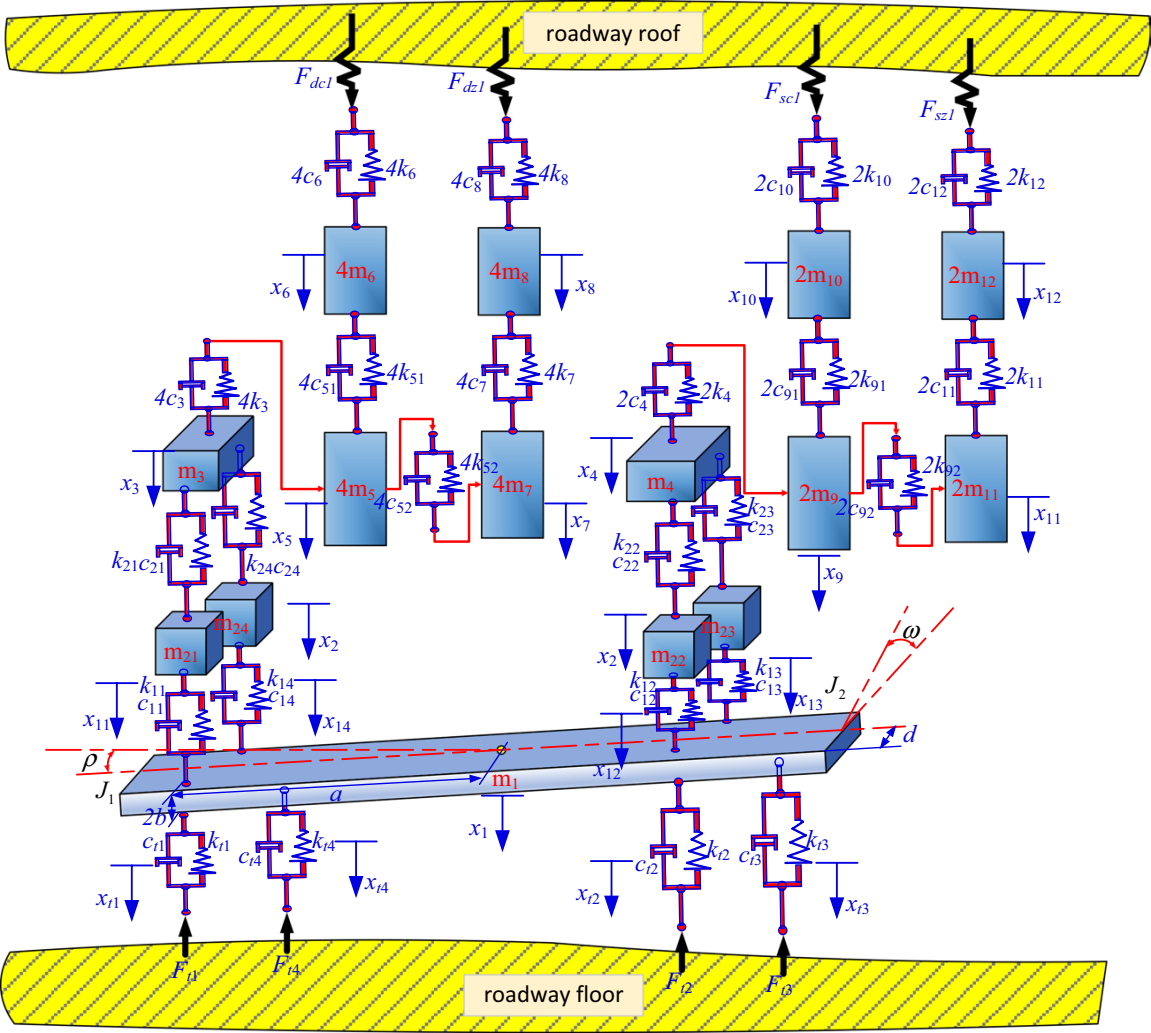
Dynamic model of anchoring system.

The main vibration type of anchoring drilling is longitudinal vibration. Therefore, taking the simultaneous working state of the top anchor drilling rig and anchor cable drilling rig as the research condition, a simplified dynamic model of the anchoring system is constructed, as shown in Fig. 2. The relevant code parameters in the dynamic model are shown in Table 1.

**Table 1:** Dynamic model parameter table.

Code	Name	Code	Name
*m*_1_	Support platform quality	*m*_21~24_	Quality of front and rear support columns
*m*_3_	Quality of top anchor beam	*m*_4_	Mass of anchor cable crossbeam
*m*_5_	Quality of top anchor drilling rig frame	*m*_6_	Quality of support plate for top anchor drilling rig
*m*_7_	Quality of power head of top anchor drilling rig	*m*_8_	Quality of top anchor drill pipe
*m*_9_	Quality of anchor cable drilling rig frame	*m*_10_	Quality of anchor cable support plate
*m*_11_	Quality of power head of anchor drilling rig	*m*_12_	Quality of anchor cable drilling rig drill pipe
*k*_11~14_ (*c*_11~14_)	Stiffness and damping between the front and rear support columns and the support platform	*k*_21~24_ (*c*_21~24_)	Stiffness and damping between front and rear support columns, top anchors, and anchor cable transverse beams
*k*_3_(*c*_3_)	Stiffness and damping between the top anchor crossbeam and the four top anchor frame	*k*_4_(*c*_4_)	Stiffness and damping between anchor cable crossbeam and anchor cable frame
*k*_51_(*c*_51_)	Stiffness and damping between the top anchor frame and the top anchor support plate	*k*_52_(*c*_52_)	Stiffness and damping between the top anchor frame and the top anchor power head
*k*_6_(*c*_6_)	Stiffness and damping between top anchor support plate and roadway roof	*k*_7_ (*c*_7_)	Stiffness and damping between the top anchor power head and the top anchor drill pipe
*k*_8_ (*c*_8_)	Stiffness and damping between top anchor drill pipe and roadway roof	*k*_91_ (*c*_91_)	Stiffness and damping between anchor cable frame and anchor cable support plate
*k*_92_ (*c*_92_)	Stiffness and damping between anchor cable frame and anchor cable power head	*k*_10_(*c*_10_)	Stiffness and damping between anchor cable support plate and roadway roof
*k*_11_(*c*_11_)	Stiffness and damping between anchor cable power head and anchor cable drill pipe	k_12_(*c*_12_)	Stiffness and damping between anchor rope drill pipe and tunnel roof
*k*_t1~ t4_ (*c*_t1~ t4_)	the equivalent stiffness and equivalent damping between legs 1–4 and the ground respectively	*a*	the distance between the center of gravity of the support platform and the leg
*b*	half the thickness of the support platform	*d*	half the width of the support platform
*J*_1_	the rotational inertia of the excavation direction of the support platform	$\rho$	the pitching vibration angle of the excavation direction of the support platform
*J*_2_	the rotational inertia of the horizontal direction of the support platform	$\varpi$	the left and right vibration angle of the horizontal direction of the support platform
*F*_*dc*1_	the reaction force of the four anchor support plate	*F*_*dz*1_	the reaction force of the four anchor drilling
*F*_*sc*1_	the reaction force of the two anchor cable support plate	*F*_*sz*1_	the drilling reaction force of the two anchor cables respectively
*x*_1_ ~ *x*_12_	the longitudinal *x* displacement of each mass block of the anchorage system in turn		

Through the analysis of the whole structure of the anchorage system, it can be seen as a multi-mass and multi-attitude system, so the Lagrange method is used to establish the dynamic model of the anchorage group system. The complete Lagrangian equation can be generally expressed as Eq. (1).

1 $$ \frac{d}{dt}\left( {\frac{\partial T}{{\partial \dot{x}_{j} }}} \right) - \frac{\partial T}{{\partial x_{j} }} + \frac{\partial V}{{\partial x_{j} }} + \frac{\partial D}{{\partial \dot{x}_{j} }} = F_{j} (t), $$

wherein, Fj(t)—external excitation force, $x_{j}$—generalized displacement, $\dot{x}_{j}$—generalized velocity, T—system kinetic energy, V—system potential energy, D—system energy dissipation function.

Using the energy method, the kinetic energy of the anchorage system is:

2 $$ \begin{gathered} T = \frac{1}{2}m_{1} \dot{x}_{1}^{2} + \frac{1}{2}(m_{21} + m_{22} + m_{23} + m_{24} )\dot{x}_{2}^{2} + \frac{1}{2}m_{3} \dot{x}_{3}^{2} + \frac{1}{2}m_{4} \dot{x}_{4}^{2} + 2m_{5} \dot{x}_{5}^{2} + 2m_{6} \dot{x}_{6}^{2} + 2m_{7} \dot{x}_{7}^{2} \hfill \\ + 2m_{8} \dot{x}_{8}^{2} + \frac{1}{2}m_{9} \dot{x}_{9}^{2} + \frac{1}{2}m_{10} \dot{x}_{10}^{2} + \frac{1}{2}m_{11} \dot{x}_{11}^{2} + \frac{1}{2}m_{12} \dot{x}_{12}^{2} + \frac{1}{2}J_{1} \dot{\rho }^{2} + \frac{1}{2}J_{2} \dot{\varpi }^{2} \hfill \\ \end{gathered} $$

The vibration of the whole machine caused by vibration is small, so it is assumed that $\sin \rho \approx \rho$,$\sin \varpi \approx \varpi$,$l_{1} = \sqrt {a^{2} + b^{2} }$,$l_{2} = \sqrt {b^{2} + d^{2} }$.

3 $$ \left\{ \begin{gathered} x_{t1} = x_{1} - l_{1} \rho - l_{2} \varpi \hfill \\ x_{t2} = x_{1} + l_{1} \rho - l_{2} \varpi \hfill \\ x_{t3} = x_{1} + l_{1} \rho + l_{2} \varpi \hfill \\ x_{t4} = x_{1} - l_{1} \rho + l_{2} \varpi \hfill \\ \end{gathered} \right.\quad \left\{ \begin{gathered} x_{11} = x_{1} + l_{1} \rho + l_{2} \varpi \hfill \\ x_{12} = x_{1} - l_{1} \rho + l_{2} \varpi \hfill \\ x_{13} = x_{1} - l_{1} \rho - l_{2} \varpi \hfill \\ x_{14} = x_{1} + l_{1} \rho - l_{2} \varpi \hfill \\ \end{gathered} \right. $$

The above formula can be simplified to Eq. (3).

4 $$ \begin{gathered} V = 2k_{t} \left\{ {(x_{1} - l_{1} \rho - l_{2} \varpi )^{2} + (x_{1} + l_{1} \rho - l_{2} \varpi )^{2} + (x_{1} + l_{1} \rho + l_{2} \varpi )^{2} + (x_{1} - l_{1} \rho + l_{2} \varpi )^{2} } \right\} \\ + \frac{1}{2}k_{1} \left\{ \begin{gathered} (x_{1} + l_{1} \rho + l_{2} \varpi - x_{2} )^{2} + (x_{1} - l_{1} \rho + l_{2} \varpi - x_{2} )^{2} + (x_{1} - l_{1} \rho - l_{2} \varpi - x_{2} )^{2} \hfill \\ + (x_{1} + l_{1} \rho - l_{2} \varpi - x_{2} )^{2} \hfill \\ \end{gathered} \right\} \\ + k_{2} (x_{2} - x_{3} )^{2} + k_{2} (x_{2} - x_{4} )^{2} + 2k_{3} (x_{3} - x_{5} )^{2} + 2k_{51} (x_{5} - x_{6} )^{2} + 2k_{6} x_{6}^{2} \\ + 2k_{52} (x_{5} - x_{7} )^{2} + 2k_{7} (x_{7} - x_{8} )^{2} + 2k_{8} x_{8}^{2} + k_{4} (x_{4} - x_{9} )^{2} + k_{91} (x_{9} - x_{10} )^{2} \\ + k_{92} (x_{9} - x_{11} )^{2} + k_{11} (x_{11} - x_{12} )^{2} + k_{12} x_{12}^{2} \\ \end{gathered} $$

The damping values in the energy dissipation equation of the anchorage system are solved with the simplified principle of component stiffness in the above potential energy, as shown in Eq. (4).

5 $$ \begin{gathered} D = 2c_{t} \left\{ {(\dot{x}_{1} - l_{1} \dot{\rho } - l_{2} \dot{\varpi })^{2} + (\dot{x}_{1} + l_{1} \dot{\rho } - l\dot{\varpi })^{2} + (\dot{x}_{1} + l_{1} \dot{\rho } + l\dot{\varpi })^{2} + (\dot{x}_{1} - l_{1} \dot{\rho } + l\dot{\varpi })^{2} } \right\} \\ + \frac{1}{2}c_{1} \left\{ \begin{gathered} (\dot{x}_{1} + l_{1} \dot{\rho } + l_{2} \dot{\varpi } - \dot{x}_{2} )^{2} + (\dot{x}_{1} - l_{1} \dot{\rho } + l_{2} \dot{\varpi } - \dot{x}_{2} )^{2} + (\dot{x}_{1} - l_{1} \dot{\rho } - l_{2} \dot{\varpi } - \dot{x}_{2} )^{2} \hfill \\ + (\dot{x}_{1} + l_{1} \dot{\rho } - l_{2} \dot{\varpi } - \dot{x}_{2} )^{2} \hfill \\ \end{gathered} \right\} \\ + c_{2} (\dot{x}_{2} - \dot{x}_{3} )^{2} + c_{2} (\dot{x}_{2} - \dot{x}_{4} )^{2} + 2c_{3} (\dot{x}_{3} - \dot{x}_{5} )^{2} + 2c_{51} (\dot{x}_{5} - \dot{x}_{6} )^{2} + 2c_{6} \dot{x}_{6}^{2} \\ + 2c_{52} (\dot{x}_{5} - \dot{x}_{7} )^{2} + 2c_{7} (\dot{x}_{7} - \dot{x}_{8} )^{2} + 2c_{8} \dot{x}_{8}^{2} + c_{4} (\dot{x}_{4} - \dot{x}_{9} )^{2} + c_{91} (\dot{x}_{9} - \dot{x}_{10} )^{2} \\ + c_{92} (\dot{x}_{9} - \dot{x}_{11} )^{2} + c_{11} (\dot{x}_{11} - \dot{x}_{12} )^{2} + c_{12} \dot{x}_{12}^{2} \\ \end{gathered} $$

Substituting Eqs. (2), (4) and (5) into Eq. (1), the following differential equations of each component can be obtained:

6 $$ \begin{gathered} m_{1} \ddot{x}_{1} = - (F_{t1} + F_{t2} + F_{t3} + F_{t4} ) - (16k_{t} x_{1} + 4k_{1} x_{1} - 4k_{1} x_{2} + 16c_{t} \dot{x}_{1} + 4c_{1} \dot{x}_{1} - 4c_{1} \dot{x}_{2} ) \hfill \\ m_{2} \ddot{x}_{2} = - (4k_{1} x_{2} - 4k_{1} x_{1} { + }4k_{2} x_{2} - 2k_{2} x_{3} - 2k_{2} x_{4} { + }4c_{1} \dot{x}_{2} - 4c_{1} \dot{x}_{1} { + }4c_{2} \dot{x}_{2} - 2c_{2} \dot{x}_{3} - 2c_{2} \dot{x}_{4} ) \hfill \\ m_{3} \ddot{x}_{3} = - \left[ {2k_{2} (x_{3} - x_{2} ) + 4k_{3} (x_{3} - x_{5} ) + 2c_{2} (\dot{x}_{3} - \dot{x}_{2} ) + 4c_{3} (\dot{x}_{3} - \dot{x}_{5} )} \right] \hfill \\ m_{4} \ddot{x}_{4} = - \left[ {2k_{2} (x_{4} - x_{2} ) + 2c_{2} (\dot{x}_{4} - \dot{x}_{2} )} \right] \hfill \\ m_{5} \ddot{x}_{5} = - \left[ {4k_{3} (x_{5} - x_{3} ) + 4k_{51} (x_{5} - x_{6} ) + 4k_{52} (x_{5} - x_{7} ) + 4c_{3} (\dot{x}_{5} - \dot{x}_{3} ) + 4c_{51} (\dot{x}_{5} - \dot{x}_{6} ) + 4c_{52} (\dot{x}_{5} - \dot{x}_{7} )} \right] \hfill \\ m_{6} \ddot{x}_{6} = F_{dc1} + F_{dc2} + F_{dc3} + F_{dc4} - \left[ {4k_{51} (x_{6} - x_{5} ) + 4k_{6} x_{6} + 4c_{51} (\dot{x}_{6} - \dot{x}_{5} ) + 4c_{6} \dot{x}_{6} } \right] \hfill \\ m_{7} \ddot{x}_{7} = - \left[ {4k_{52} (x_{7} - x_{5} ) + 4k_{7} (x_{7} - x_{8} ) + 4c_{52} (\dot{x}_{7} - \dot{x}_{5} ) + 4c_{7} (\dot{x}_{7} - \dot{x}_{8} )} \right] \hfill \\ m_{8} \ddot{x}_{8} = F_{dz1} + F_{dz2} + F_{dz3} + F_{dz4} - \left[ {4k_{7} (x_{8} - x_{7} ) + 4k_{8} x_{8} + 4c_{7} (\dot{x}_{8} - \dot{x}_{7} ) + 4c_{8} \dot{x}_{8} } \right] \hfill \\ m_{9} \ddot{x}_{9} = - \left[ {2k_{4} (x_{9} - x_{4} ) + 2k_{91} (x_{9} - x_{10} ) + 2k_{92} (x_{9} - x_{11} ) + 2c_{4} (\dot{x}_{9} - \dot{x}_{4} ) + 2c_{91} (\dot{x}_{9} - \dot{x}_{10} ) + 2c_{92} (\dot{x}_{9} - \dot{x}_{11} )} \right] \hfill \\ m_{10} \ddot{x}_{10} = F_{sc1} + F_{sc2} - \left[ {2k_{91} (x_{10} - x_{9} ) + 2c_{91} (\dot{x}_{10} - \dot{x}_{9} )} \right] \hfill \\ m_{11} \ddot{x}_{11} = - \left[ {2k_{92} (x_{11} - x_{9} ) + 2k_{11} (x_{11} - x_{12} ) + 2c_{92} (\dot{x}_{11} - \dot{x}_{9} ) + 2c_{11} (\dot{x}_{11} - \dot{x}_{12} )} \right] \hfill \\ m_{12} \ddot{x}_{12} = F_{sz1} + F_{sz2} - \left[ {2k_{11} (x_{12} - x_{11} ) + 2k_{12} x_{12} + 2c_{11} (\dot{x}_{12} - \dot{x}_{11} ) + 2c_{12} \dot{x}_{12} } \right] \hfill \\ J_{1} \ddot{\rho } = - (16l_{1}^{2} k_{t} \rho + 4l_{1}^{2} k_{1} \rho + 16l_{1}^{2} c_{t} \dot{\rho } + 4l_{1}^{2} c_{1} \dot{\rho }) \hfill \\ J_{2} \ddot{\varpi } = - (16l_{2}^{2} k_{t} \varpi + 4l_{2}^{2} k_{1} \varpi + 16l_{2}^{2} c_{t} \dot{\varpi } + 4l_{2}^{2} c_{1} \dot{\varpi }) \hfill \\ \end{gathered} $$

## Rigid–flexible coupling dynamics solution of the anchorage system

### Modal characteristic analysis of anchorage system

**Figure 3:**
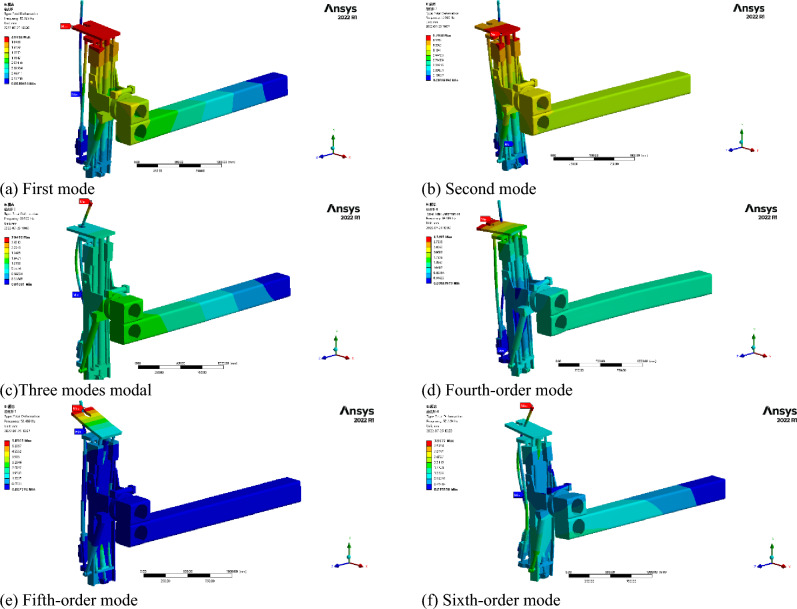
Modal analysis of anchoring drill.

The structure of the anchorage system is complex. In order to simplify the system calculation, the modal influence law of key components of the anchorage system is studied under the constraint of actual anchorage drilling conditions. The modal-frequency-deformation diagram of the following key components was obtained by using Ansys finite element analysis software. The resonance frequency range of the key components of the anchoring system under different modal orders was studied. Considering the external excitation frequency range of the anchoring system, the 1–6 order modal characteristics of the key drilling rig components were selected for analysis. The obtained modal diagram and eigenvalue diagram are shown in Fig. 3 and Table 2. It can be seen that the key components of the anchoring system have resonance effects at frequencies of 10.325 Hz, 13.905 Hz, 29.532 Hz, 39.619 Hz, 51.489 Hz, and 57.669 Hz. The maximum amplitude is 5.86 mm. The modal analysis law of the anchorage system can provide a theoretical comparison for the later field test.

**Table 2:** Modal analysis of anchor system.

Modal order	Frequency/HZ	Deformation/mm
1	10.325	2.08
2	13.905	1.79
3	29.532	2.95
4	39.619	3.12
5	51.489	5.86
6	57.669	3.97

In order to achieve the goal of accuracy and low computational complexity of the dynamic analysis of the anchoring system, this paper uses the rigid-flexible coupling multi-body dynamics solution method to flexibly process the actual deformed components in the anchoring system. The components are divided into a combination of multiple rigid components and flexible components to more accurately simulate the mechanical characteristics of the actual anchoring drilling process. This method focuses on the analysis of the vibration characteristics of the major components of the whole machine overtime under the external load during the drilling process, which provides an important reference for the stability of the whole machine.

**Figure 4:**
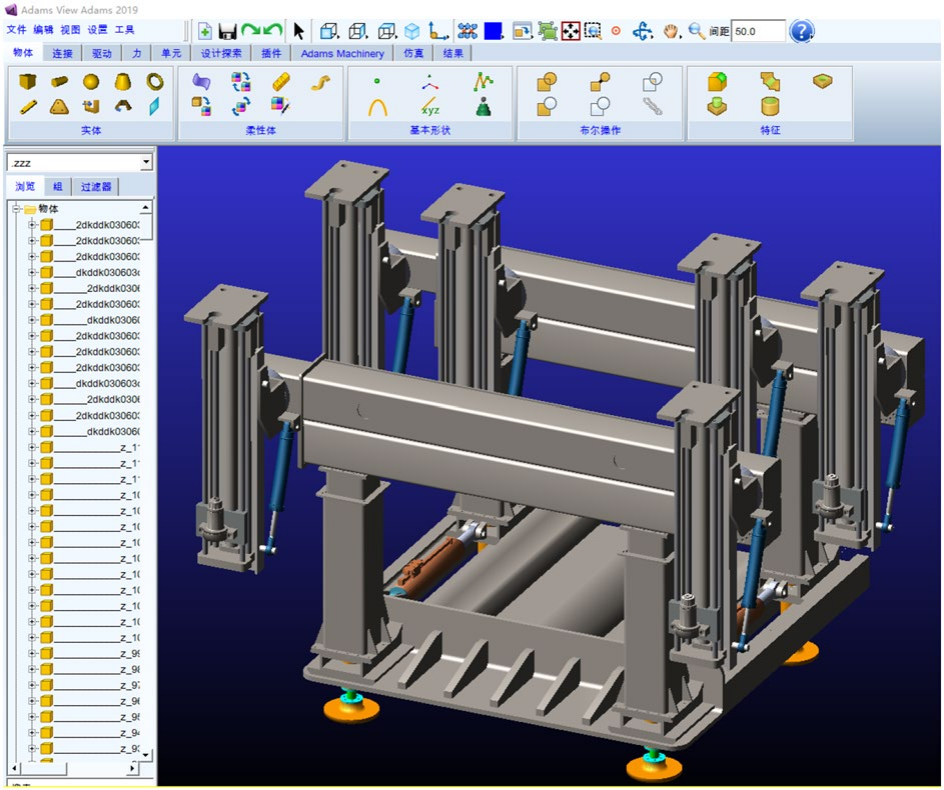
Rigid flexible coupling dynamics model.

**Figure 5:**
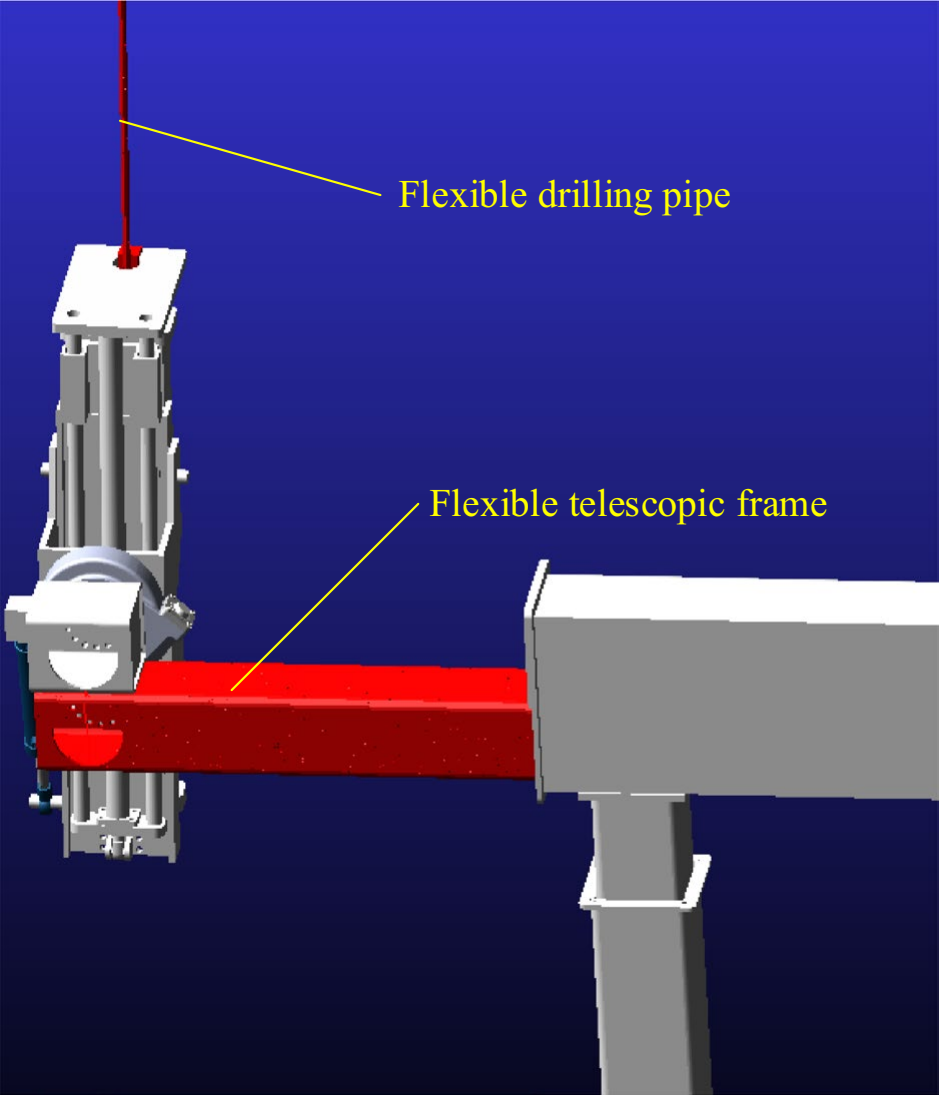
Flexible body component display.

Through Solidworks' three-dimensional modeling software, the model data of the anchoring system drilling rig is transmitted to Adams, and the material properties, mutual restraint relationship (i.e. motion pair), drive, gravity acceleration, and external excitation force and torque of the drilling process are applied in turn. Considering the cantilever state of the telescopic frame and the large deformation vibration characteristics of the drill pipe during the operation of the anchoring system, the drill pipe and the telescopic frame are rigidly flexible. Based on the large stiffness and small deformation of the telescopic frame and the small size and large deformation characteristics of the drill pipe, the vibration characteristics and mechanical characteristics of each component of the unit are analyzed. The corresponding rigid-flexible coupling dynamic model is established, as shown in Figs. 4 and 5.

### Dynamic analysis of anchorage group system under retraction/extension of cantilever

#### Vibration analysis of drill pipe in cantilever retraction/extension state

**Figure 6:**
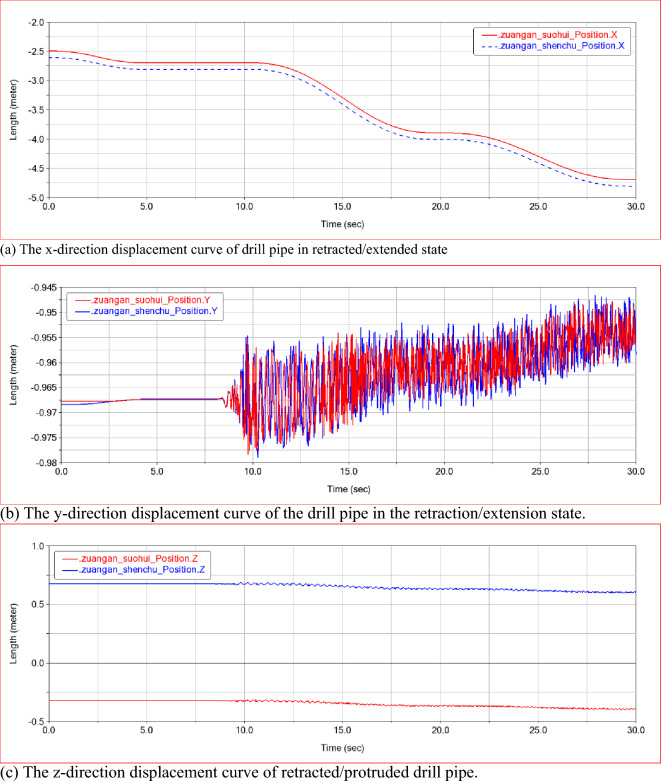
Drill pipe displacement curve in retracted/extended state.

Based on the characteristics of small diameter and large deformation of drill pipe, the movement trend of drill pipe in the axial direction is approximately the same during the initial position adjustment and drilling process, as shown in Fig. 6a. As shown in Fig. 6b. In the following figure, 'suohui' stands for 'retracted' and 'shenchu' stands for 'extended'. In the first 0 ~ 10 s, the y-direction displacement of the drill pipe in the retraction / extension state is roughly similar, basically maintaining the same level. In the process of 10 ~ 30 s drilling rig position adjustment and drilling, the y-direction vibration displacement of the drill pipe in the extension state of the telescopic frame is obviously larger than that in the retraction state of the telescopic frame, and it can be concluded that the vibration radius of the drill pipe in different stages of the multi-stage feed process of the drilling rig is significantly different, among which the vibration radius of the drill pipe in the one-time feed process of the drilling rig is the largest, about 0.0125 m. In the extension direction of the telescopic frame, the vibration of the drill pipe is small, and the movement and vibration trends under the two working conditions are similar. As shown in Fig. 6c, the displacement of the drill pipe in the z-direction of the retracted / extended state is not obvious in the first 0–10 s. At 10–30 s, the drill pipe has a small amplitude fluctuation in the z-direction displacement. Because the drilling rig support plate is equipped with a gripper to reduce the vibration of the drill pipe, it can meet the actual anchoring drilling requirements.

#### Analysis of drill pipe vibration acceleration law

**Figure 7:**
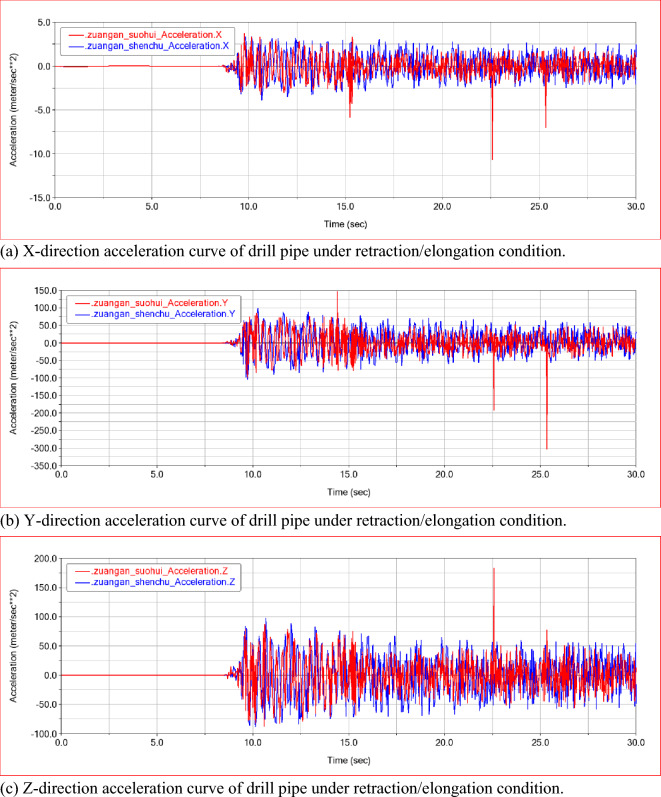
Acceleration curve of drill pipe in retracted/extended state.

By analyzing the law of the acceleration of the drill pipe in each direction during the drilling process, it can be seen that the acceleration of the drill pipe tends to be stable under the operating conditions of the 0–10 s rig adjustment attitude. In the drilling process of 10–30 s, the acceleration of each direction fluctuates within a certain range due to the influence of drilling resistance. The trend of the acceleration curve of the drill pipe under the extended and retracted state of the telescopic frame is basically the same, but the vibration range under the extended state of the telescopic frame is larger. As shown in Fig. 7a–c, there are three instantaneous peak responses of the three-way acceleration of the drill pipe during the drilling process under the retracted state of the drill pipe, but the response time is very short, which does not affect the overall vibration law. In general, the vibration acceleration of the drill pipe under the extended state of the telescopic frame is still stronger than that under the retracted state, and the maximum value of the three-way vibration acceleration of the drill pipe is 4 m/s^2^, 100 m/s^2^, 80 m/s^2^.

#### Power spectral density analysis of drill pipe vibration

**Figure 8:**
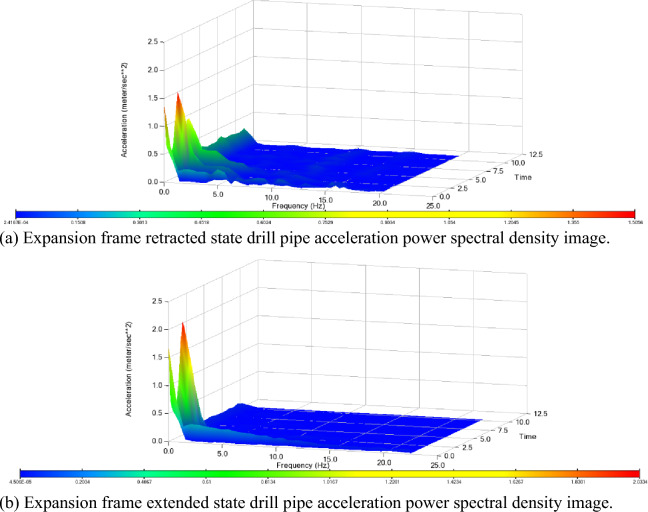
Drill pipe acceleration power spectral density image.

Using the FFT transform function in the post-processing module PostProcessor to perform fast Fourier transform processing on the time-domain acceleration curves in the three directions of the drill pipe in the extended and retracted states of the above telescopic frame, the corresponding power spectral density images can be obtained. The results are shown in Fig. 8. By analyzing the power spectral density images of the two states, it can be seen that the load frequency applied to the drill pipe is lower than 25 Hz. When the telescopic frame is extended, the vibration acceleration of the drill pipe is larger at 1.1 Hz, 2 Hz, and 5 Hz. When the telescopic frame is retracted, the vibration acceleration of the drill pipe is larger at 1.1 Hz and 2 Hz. The distribution density of the vibration response in the retracted state is less than that in the extended state, but the maximum response acceleration of the drill pipe in the retracted state (2.0 m/s^2^) is greater than that in the retracted state (1.5 m/s^2^). Combined with the modal natural frequency analysis of the drill pipe, the minimum natural frequency of the drill pipe is greater than 5 Hz, which will not affect the drilling efficiency.

## Analysis of influence factors of drill pipe drilling different surrounding rock vibration

**Figure 9:**
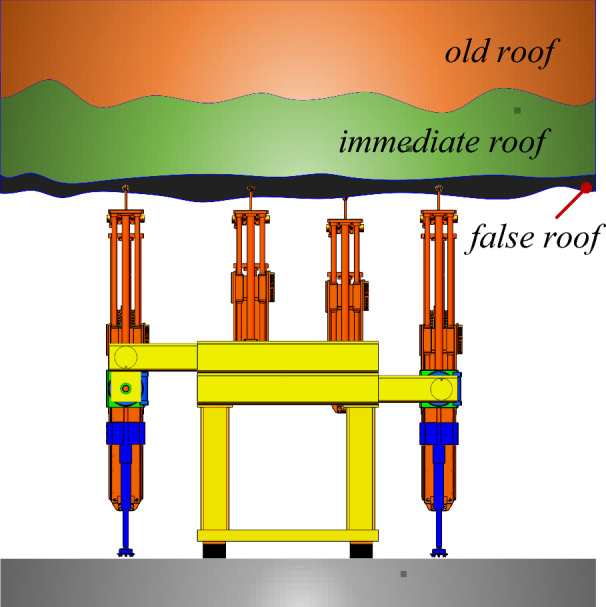
Diagram of anchoring drilling coal rock.

The roof of the coal mine roadway can be divided into the false roof, direct roof, and old roof according to different rock properties. In the actual drilling process, the drill pipe needs to drill into the above three rock strata to realize the anchoring effect, as shown in Fig. 9. In order to study the mechanism of vibration characteristics of drill pipe under different rock properties, Abaqus mechanical analysis software was used to simulate the vibration response law of drill pipe when drilling different surrounding rocks.

The material properties of fine sandstone, siltstone, and mudstone and the material properties of drilling tools are shown in Table 3. Different rock material property parameters are assigned to rock specimens in Abaqus, and the plastic failure of rock adopts the Drucker Prager yield criterion. Peak stress can represent rock hardness.

**Table 3:** Material parameter.

Material	Density (kg/m^3^)	Peak stress (Mpa)	Peak axial strain (%)	Young's modulus (Gpa)	Poisson ratio
Old roof	2710.17	94.63	0.646	23.07	0.09
Immediate roof	2238.84	54.45	0.591	18.06	0.16
False roof	2539.16	30.05	0.638	9.19	0.07
42 Cr Mo alloy	7850	930		210	0.33

To simplify the key analysis of the model, the following basic assumptions are made:

1. During the drilling process, the drilling trajectory is well controlled, and the drill bit is drilled perpendicular to the roadway roof, without considering the friction between the drill rod and the hole wall.
2. Due to the high hardness and strength of the drill bit of the anchor rod drill relative to the rock mass of the roadway roof, it is assumed that the drill bit is a rigid body.
3. After the failure of rock unit drilling, the problem of repeated crushing is no longer considered, and the broken rock unit will no longer affect subsequent drilling work.

Use 3D modeling software to finely model the roadway roof, mining two wing drill bits, and drill rods. During the modeling process, efforts should be made to minimize the refinement of complex points such as small rounded corners and chamfers. The size of the drill bit and the size of the simulated roadway should roughly follow the requirement that the distance between the drilling center and the boundary should be greater than 5 times the diameter of the drilling head. Considering the subsequent synchronous operation process of multiple drilling machines, the roadway roof model should be set to an equal proportion strip shape; Import the above model into the mechanical simulation software Abaqus, and when performing mesh partitioning operations, consider that the simulation drilling simulation is a dynamic display simulation. For complex components (drill bits), the mesh type needs to be set to tetrahedral format. To ensure sufficient contact between the drill bit and the coal wall and simulate actual drilling operations, in the early stage of constructing the mesh of the roadway roof, the plane mesh division function is used to locally encrypt the mesh with the drilling area. Set the drill bit as a rigid body, with a binding connection between it and the drill pipe, and use a surface to surface contact relationship between the drill bit and the dense body in the middle of the coal wall.

**Figure 10:**
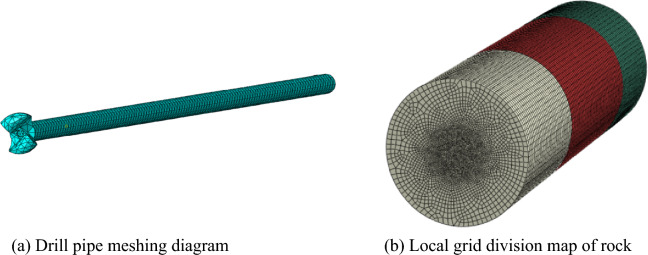
Key component grid division diagram.

**Figure 11:**
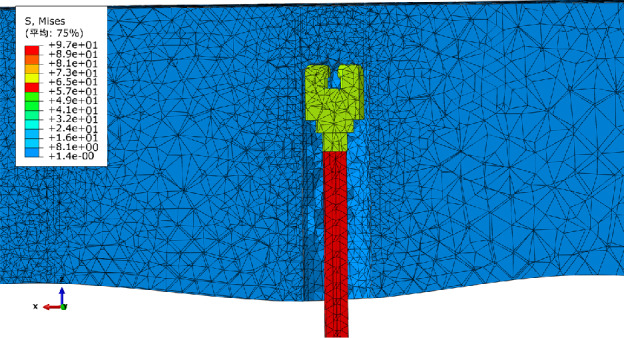
Drilling simulation diagram.

Considering the stress area of the drill pipe and rock during the drilling process, the drill pipe and rock are divided into local refined grids, as shown in Fig. 10. The drill pipe and rock are further constrained and driven to simulate the actual drilling conditions. Three sets of drilling tests are carried out by changing the properties of the rock, and the drilling vibration law of the drill pipe under different surrounding rock properties is obtained. The drilling simulation diagram is shown in Fig. 11.

**Figure 12:**
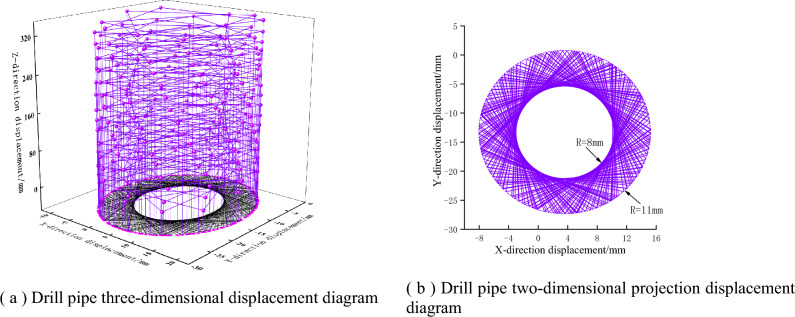
Vibration displacement response diagram of drill pipe under false roof drilling condition.

**Figure 13:**
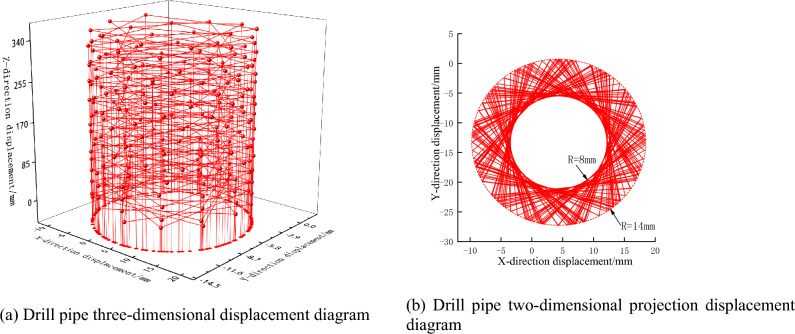
Vibration displacement response diagram of drill pipe under direct roof drilling condition.

**Figure 14:**
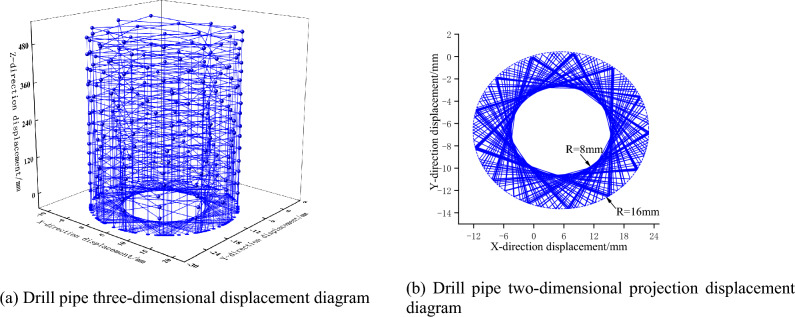
Drill pipe vibration displacement response diagram under old roof drilling condition.

By drilling three working conditions of false roof, direct roof, and old roof respectively, applying the same constraints and driving conditions, the vibration displacement law of drill pipe under different coal and rock conditions is analyzed. As shown in Figs. 12, 13 and 14, with the continuous drilling of the drill pipe in the Z direction, the lateral displacement of the drill pipe shows a certain range of vibration fluctuations. When drilling the above three working conditions, the lateral vibration radius of the drill pipe is 5 mm, 6 mm, and 8 mm respectively. It can be seen that with the increase in the hardness of the coal rock, the lateral vibration displacement of the drill pipe is larger, and the drilling effect of the response drilling is worse.

## Experimental Verification

**Figure 15:**
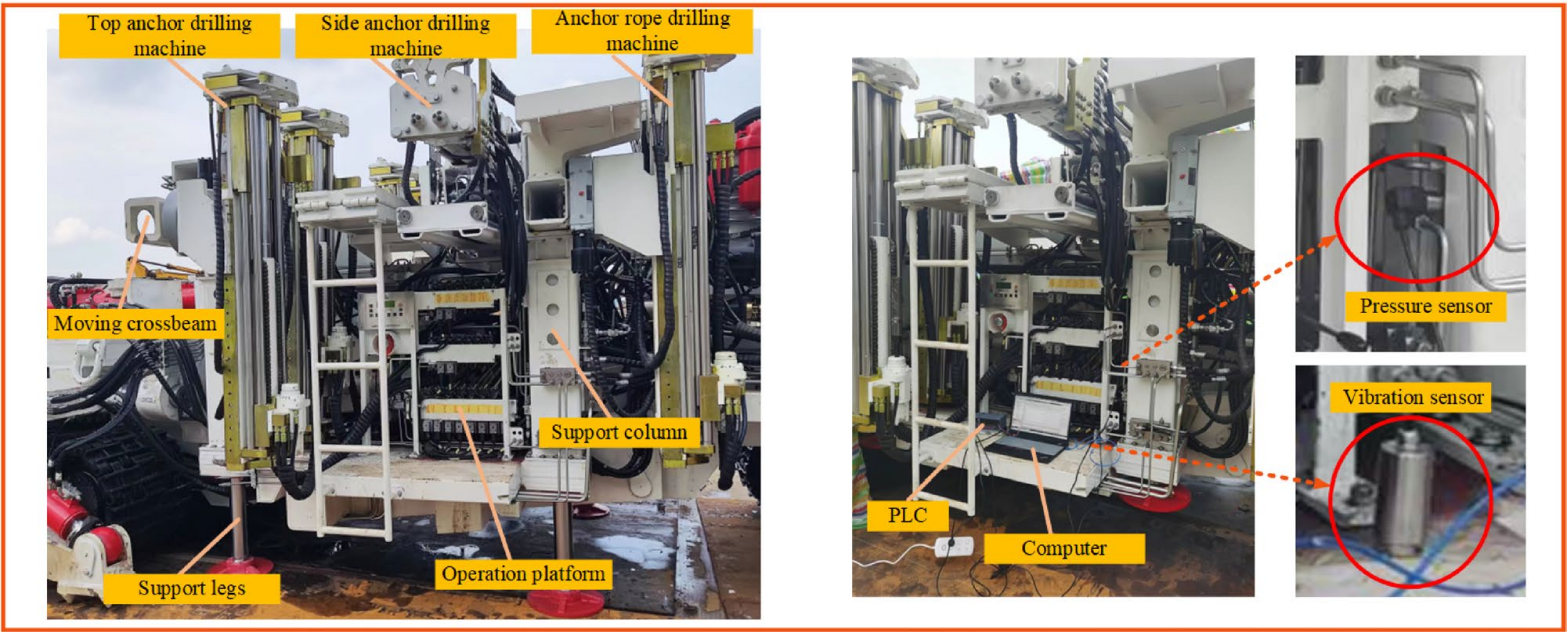
Physical diagram of test prototype.

In order to obtain the vibration characteristic mechanism of the anchoring system, an experimental prototype of the anchoring unit was designed to simulate the field coal rock properties for the anchoring drilling test, as shown in Fig. 15. Because the standing wave wavelength of high-frequency modes is short, more monitoring response points are needed to describe these modes properly. The test platform is composed of a drilling body, support platform, lifting platform, 16-channel DH5922 N dynamic signal test system, DH5857 charge regulator, computer, and more than 10 DH311E three-way acceleration sensors. The modal and vibration law of the key components of the anchorage system is analyzed in turn.

### System modal analysis

The test results are collected and analyzed by a 16-channel data acquisition system. The signal at this time is a time-domain signal. It is necessary to identify the modal parameters of the time-domain signal. The multi-reference point least squares complex frequency domain method is used to extract the modal parameters of the vibration system.

**Figure 16:**
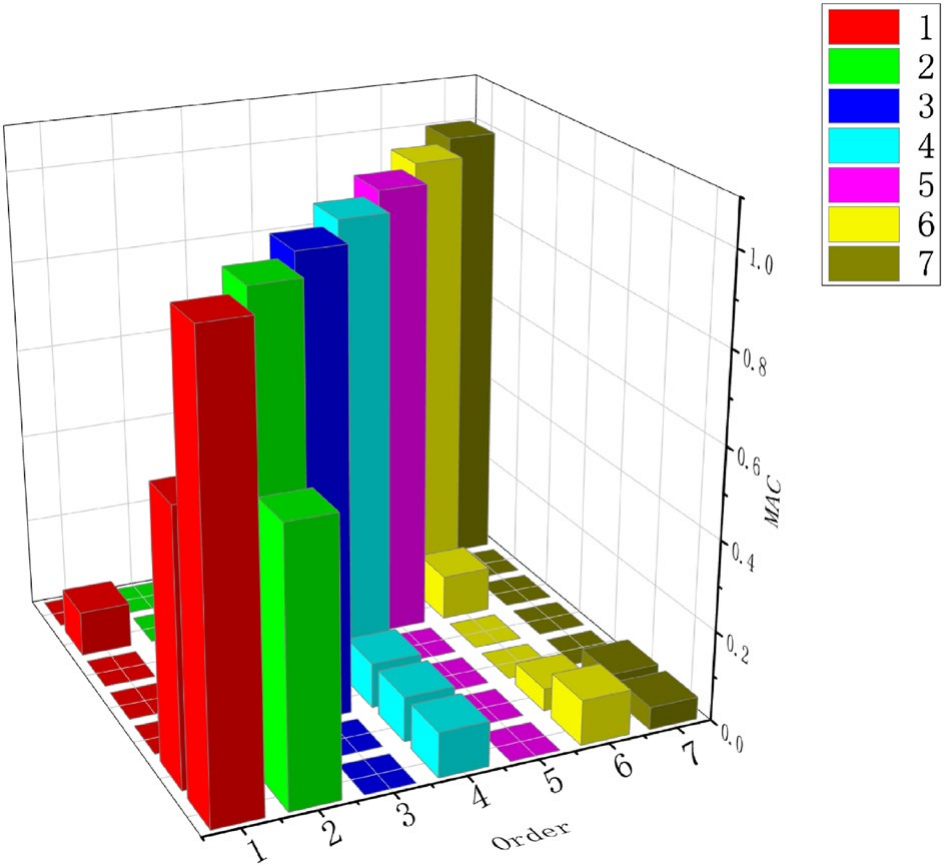
Modal MAC value.

The frequency values of the anchoring system under different orders measured in the field and the frequency values obtained by the theory are shown in Table 4 below. The modal analysis experimental results are verified by the modal determination criterion MAC value method. If the nth-order mode is exactly the same as itself, the MAC value is equal to 1. If the difference between the two is large, the MAC value is close to 0. The obtained MAC value is shown in Fig. 16. Except between the modes, the MAC values between the modes are basically within 0.7, and most of them are within 0.2, indicating that the correlation of each mode is low and the orthogonality is good. According to the principle of vibration mechanics, the natural vibration modes of different orders are orthogonal to each other, so the modes obtained from the test are correct.

**Table 4:** Modal data table of anchorage system.

Rank	Actual frequency	Theoretic frequency
1	10.131	10.325
2	13.783	13.905
3	28.489	29.532
4	40.112	39.619
5	50.892	51.489
6	58.732	57.669

When the frequency range of drilling disturbance varies from 250 to 300 rad/s, the intermediate value is around 275 rad/s, i.e. 43.80 Hz, while the fourth natural frequency obtained from the test is 40.112 Hz, so the system will not resonate. The modal parameters of other orders of the anchor group are also obtained in the modal experiment. However, because the natural frequencies of other orders are different from the disturbance frequencies of the anchor drilling, resonance will not be caused in these frequency ranges.

### Analysis of drill pipe vibration law in simulated drilling process

**Figure 17:**
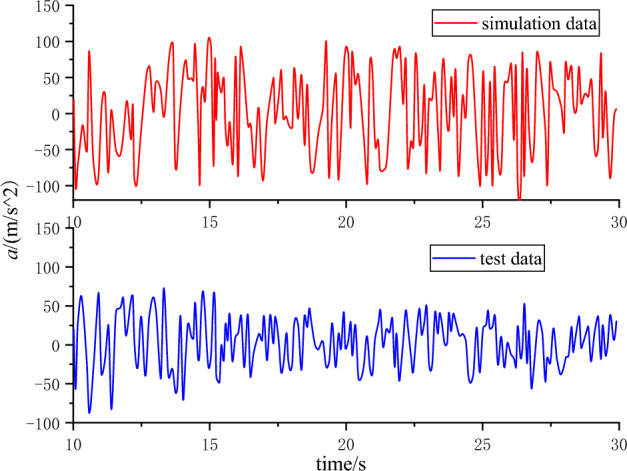
Drill pipe vibration response test and simulation comparison.

Through the dynamic monitoring signal system, the vibration response curve of the monitoring point at the drill pipe is obtained. This paper mainly studies the longitudinal vibration of the drill pipe. For this reason, the experimentally measured data is compared with the above simulation data. As shown in Fig. 17, the time domain response diagram of the drill pipe vibration obtained by the simulation analysis is not much different from the overall fluctuation range of the data obtained by the test. Considering the influence of different rig layouts and structural stiffness, the response amplitude obtained by the test is slightly larger than the simulation amplitude, which basically verifies the accuracy of the simulation data.

## Conclusion

1. Considering the simultaneous anchoring operation of multiple drilling rigs, a dynamic model of the anchoring system is constructed using the Lagrange equation, and the vibration differential equations of each component are obtained;
2. Using Ansys finite element analysis software to obtain the sixth order modal law of the anchoring system drilling rig components, providing a theoretical basis for the later anchoring system modal test;
3. Using Adams dynamics software to solve the rigid flexible coupling dynamics of the anchoring group, the beam expansion frame is a short body large-span structure in the extended state, and the vibration displacement, vibration acceleration and other parameters of the drill pipe are significantly greater than those in the retracted state, indicating a significant vibration effect;
4. Using Abaqus to simulate the vibration response of drill pipe drilling under different rock properties, the results show that the greater the hardness of the drill pipe drilling into the rock, the stronger the vibration response of the drill pipe.

By conducting drilling experiments on the prototype of the anchoring system, the theoretical reliability of the drilling vibration laws of key drilling rig components is verified. The relevant theoretical achievements can provide a theoretical basis for the study of the stability of drilling vibration in the anchoring system.

## Data Availability

All data generated or analyzed during this study are included in this published article. Request for more details to the corresponding author.
